# Potential Off-Target Effect of Gilteritinib With Venetoclax Decreases Tumor Burden for Patients With Relapsed/Refractory Wild-Type *FLT3* Acute Myeloid Leukemia/Myelodysplastic Neoplasms

**DOI:** 10.1155/crh/4335095

**Published:** 2025-10-29

**Authors:** Shuting Chang, Zhijuan Pan, Yiqun Zhang, Ying Zhang, Jiajia Sun, Yanru Guo, Xinlei Guo, Zhiping Guo

**Affiliations:** Department of Hematology, Peking University First Hospital Taiyuan Branch (Taiyuan Central Hospital), The Ninth Clinical Medical College of Shanxi Medical University, Taiyuan 030006, China

**Keywords:** acute myeloid leukemia, gilteritinib, myelodysplastic neoplasms, refractory, relapsed, venetoclax, wild-type *FLT3*

## Abstract

Many tyrosine kinase inhibitors show nonspecific activity against multiple kinases, causing off-target effects when used in a broad patient population. This study evaluated the effectiveness of gilteritinib combined with venetoclax in patients with relapsed/refractory (R/R) acute myeloid leukemia (AML) or myelodysplastic neoplasms (MDS) with wild-type *FLT3*, who currently lack targeted therapy. After a 28-day cycle of venetoclax–gilteritinib therapy, one patient with R/R AML and other genetic alterations achieved minimal residual disease (MRD)–positive complete remission (CR) with incomplete hematologic recovery (CRi). Another patient with R/R *ASXL1*-mutated MDS/AML achieved morphologic leukemia-free state (MLFS) after one cycle, but cytopenias persisted across two cycles. A patient with R/R *TP53*-mutated AML related to myelodysplasia did not respond (NR) after two cycles, although the blast percentage in bone marrow (BM) and peripheral blood (PB) decreased by 50%. In a patient with R/R AML carrying an in-frame bZIP-mutated *CEBPA*, NR and disease progression occurred after one cycle, but elevated white blood cell (WBC) counts declined after treatment initiation and lasted for 2 weeks. These findings suggest that combining gilteritinib with venetoclax may reduce tumor burden in R/R AML/MDS patients with wild-type *FLT3*.

## 1. Introduction

Myelodysplastic neoplasms (MDS) and acute myeloid leukemia (AML) are heterogeneous blood cancers defined by complex molecular and genetic abnormalities that affect prognosis and treatment response. Genetic analyses have shown that both MDS and AML patients frequently harbor recurrent mutations [1]. In addition, aside from de novo AML, a large proportion of MDS patients progress to AML [2]. Recent progress in targeted therapies directed at specific mutations in MDS and AML has improved overall survival [3]. However, the prognosis for patients with relapsed/refractory (R/R) disease remains poor, and treatment options are limited [4]. Many R/R AML/MDS patients do not have identifiable mutations that are suitable for targeted therapy.

Protein tyrosine kinases (PTKs) play key roles in transmitting signals that drive cell proliferation, making them common oncogenic drivers in human cancers [5]. Within this family, the fms-like tyrosine kinase receptor (FLT3), FMS, KIT, and platelet-derived growth factor receptor (PDGFR) share considerable sequence similarity [6]. Many tyrosine kinase inhibitors (TKIs) display nonspecific activity across these kinases, leading to off-target effects when used broadly. For example, dasatinib, an oral BCR-ABL TKI, is approved for chronic myeloid leukemia [7], Philadelphia-positive leukemia [8], chronic myeloproliferative disorders with *PDGFRβ* rearrangements [9], some mast cell leukemias (MCL) with noncanonical *KIT* mutations [10], and *KIT*-positive AML [11]. Dasatinib may also be useful as an antileukemic therapy for *FLT3*-internal tandem duplication (*ITD*)-positive AML and *PTPN11*-mutated AML [12]. Ibrutinib, an oral inhibitor of Bruton tyrosine kinase (BTK), is used to treat mantle cell lymphoma, chronic lymphocytic leukemia, and Waldenström macroglobulinemia [13, 14]. Ex vivo analyses have shown that ibrutinib has antileukemic activity in AML with concurrent *NPM1*, *FLT3*, and *DNMT3A* mutations [15]. In addition, ibrutinib combined with decitabine has shown significant clinical activity in *TP53*-mutated AML and high-risk MDS [16]. Midostaurin, an inhibitor of several Type III tyrosine kinase receptors, is approved for newly diagnosed adult patients with *FLT3*-mutated AML and advanced systemic mastocytosis (SM) [17, 18], especially MCL with *KIT* D816 mutations [10]. Midostaurin also represents a promising option for relapsed t(8; 21) AML with *KIT* mutations [19].

Gilteritinib is a second-generation Type I TKI that primarily targets FLT3 and AXL, both of which are oncogenic kinases [20]. Gilteritinib monotherapy has shown strong antileukemic activity in R/R *FLT3*-mutated AML [21–23]. Furthermore, gilteritinib combined with venetoclax, a selective inhibitor of B-cell lymphoma 2 (BCL-2), has achieved high response rates in R/R *FLT3*-mutated AML [24]. Preclinical studies suggest that the synergistic effect of gilteritinib with venetoclax also extends to *FLT3*-wild-type cells, possibly due to gilteritinib's affinity for AXL [25]. However, clinical evidence for this approach is still lacking. This study therefore evaluated the efficacy of gilteritinib combined with venetoclax in patients with R/R AML or MDS and wild-type *FLT3*.

## 2. Materials and Methods

### 2.1. Inclusion Criteria

Patients diagnosed with AML/MDS were confirmed according to the World Health Organization (WHO) 2022 guidelines [26]. Before treatment initiation, patients underwent re-assessment using next-generation sequencing (NGS) of RNA from bone marrow (BM) aspirates. Non-germline mutations were validated through analysis of oral mucosal cells. Eligible patients had wild-type *FLT3* (*FLT*3^WT^). R/R disease was defined according to the 2022 European Leukemia Network (ELN) criteria for AML [27] and the 2023 International Working Group (IWG) response criteria for MDS [28].

This study was approved by the Ethics Committee of Peking University First Hospital Taiyuan Branch (Taiyuan Central Hospital of Shanxi Medical University). Written informed consent was obtained from the patient's family in accordance with the Declaration of Helsinki.

### 2.2. Case Presentation

#### 2.2.1. Case 1

A 59-year-old female was diagnosed with AML harboring an in-frame basic leucine zipper (bZIP) *CEBPA* mutation, classified as favorable risk, with a karyotype of 46, XX. She initially received venetoclax–azacitidine induction, achieving complete remission (CR), followed by two additional cycles of the same regimen and one cycle of DCAG consolidation. Shortly after consolidation, the disease relapsed. Salvage CLAG chemotherapy was administered, resulting in a second CR. However, 2 months later, a relapse occurred with BM blasts of 53%. The patient then received the standard “3 + 7” regimen, consisting of IA induction followed by two cycles of MA induction. Post-treatment BM analysis revealed 59% blasts, with symptoms of fatigue and nausea. After three months of best supportive care (BSC), the white blood cell (WBC) count increased to 137.78 × 10^9^/L. Peripheral blood (PB) and BM analyses showed blast percentages of 92% and 76.5%, respectively. An RNA-based NGS panel detected no *FLT3* mutation but confirmed biallelic *CEBPA* mutations and several additional alterations: *ARID1A* p.Ala246Val (30.23%); *ATM* p.Pro2512Arg (13.79%); *DNM2* p.Ser534_Tyr537del (33.05%); *EP300* p.Pro1971His (43.37%); *FGFR3* p.Arg116His (30.77%); *RAF1* p.Ala119Val (48.21%); and *SAMHD1* p.Arg333His (55.56%). Non-germline mutations were validated by analysis of oral mucosal cells.

#### 2.2.2. Case 2

A 62-year-old male was initially diagnosed with MDS with low blasts (MDS-LB). Chromosomal analysis revealed a complex karyotype [40–45, XY, −2, del(5) (q13q33), −7, der(10)t(2; 10) (q21; q25), add(12) (p11.2), −13, +1–3mar], placing him in the high-risk category according to the Revised International Prognostic Scoring System (IPSS-R). He first received two cycles of decitabine induction but progressed to MDS with increased blasts (MDS-IB2). Treatment with DCAG achieved partial remission (PR). Progression occurred after three cycles of venetoclax–azacitidine, followed by DHAG therapy. Within 15 months of diagnosis, he progressed to AML, presenting with bone pain. BM aspirate showed 63% blasts on smear and 27.5% by multiparameter flow cytometry. RNA-based NGS identified a *TP53* R248Q mutation with a frequency of 86.67% and a *PTPN11* T507K variant with a frequency of 69.09%. No *FLT3* mutation was detected.

#### 2.2.3. Case 3

A 63-year-old male was diagnosed with MDS with low blasts (MDS-LB). Chromosomal analysis revealed a normal karyotype. He was classified as high risk according to the IPSS-R. The patient initially received four cycles of decitabine and achieved CR. After 8 months of observation, cytopenia reappeared. Subsequent azacitidine monotherapy for four cycles resulted in another CR. However, 6 months later, disease progression occurred, and six cycles of decitabine proved ineffective. After 6 months of BSC, the patient developed fatigue, nausea, and weight loss. Repeat BM aspiration showed 10% blasts on smear and 4.81% minimal residual disease (MRD) by flow cytometry, consistent with MDS/AML. RNA sequencing identified a nonsense mutation in additional sex combs-like 1 (*ASXL1*) and wild-type *FLT3*.

#### 2.2.4. Case 4

A 65-year-old female was diagnosed with AML with other defined genetic alterations. Her karyotype was 46, XX. She achieved CR with MRD negativity (CR_MRD-_) 28 days after venetoclax–azacitidine therapy, followed by six cycles of venetoclax–azacitidine consolidation. Eight months later, MRD became positive (2.63%) by flow cytometry. BM smear revealed 6% blasts with Auer rods, indicating hematologic relapse. Gene mutation testing of a BM sample showed a *CEBPA* mutation at 6.3% (single mutation outside the bZIP region) without *FLT3* mutation.

**Figure 1:**
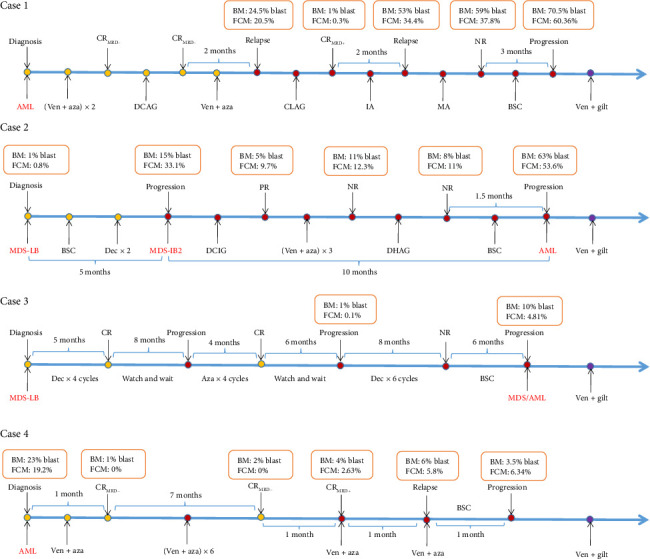
Clinical course of the patients. CR: complete remission; NR: no response; PR: partial remission; DCAG: decitabine 20 mg/m^2^/day for 5 days, low-dose cytarabine 10 mg/m^2^ every 12 h on Days 1–14, aclarubicin 20 mg/day on Days 1–4, and G-CSF 200 μg/m^2^ on Days 0–14; CLAG: cladribine 5 mg/m^2^ on Days 1–5, cytarabine 2 g/m^2^ on Days 1–5, G-CSF 5 μg/kg on Days 0–5; IA: idarubicin 10 mg/m^2^ for 3 days and cytarabine 100 mg/m^2^ for 7 days; MA: mitoxantrone 8 mg/m^2^ for 3 days and cytarabine 100 mg/m^2^ for 7 days; decitabine: 20 mg/m^2^/day for 5 days; DCAG: low-dose cytarabine 10 mg/m^2^ every 12 h on Days 1–14, aclarubicin 20 mg/day on Days 1–4, and G-CSF 200 μg/m^2^ on Days 1–14; DHAG: low-dose homoharringtonine 1 mg/m^2^ on Days 1–14, low-dose cytarabine 10 mg/m^2^ every 12 h on Days 1–14, and G-CSF 200 μg/m^2^ on Days 1–14; azacitidine: 100 mg/m^2^ × 7 days.

The treatment flow diagram is shown in Figure 1.

### 2.3. Treatment

Hydroxyurea was permitted for cytoreduction and control of WBC counts at the initiation of study drug treatment. Gilteritinib was administered orally once daily from Day 1 at doses of 80 or 120 mg for dose escalation [21]. Venetoclax was given orally once daily starting on Day 1 with a 3-day dose ramp-up (100 mg on Day 1, 200 mg on Day 2, and 400 mg on Days 3–28), and continued at 400 mg in subsequent cycles. The combination of gilteritinib and venetoclax was delivered in 28-day cycles until disease progression. All patients received red blood cell (RBC) and platelet transfusions to manage anemia and thrombocytopenia. Growth factor support was allowed after BM blasts decreased to < 5% or in cases of neutropenic sepsis. Prophylaxis for tumor lysis syndrome included hydration, uric acid–lowering agents, and regular monitoring of blood chemistry. BM evaluation was performed on Day 28 of each cycle, and PB blast percentage was monitored twice weekly in patients with a high proportion of circulating blasts before treatment.

## 3. Results

### 3.1. Response

**Figure 2:**
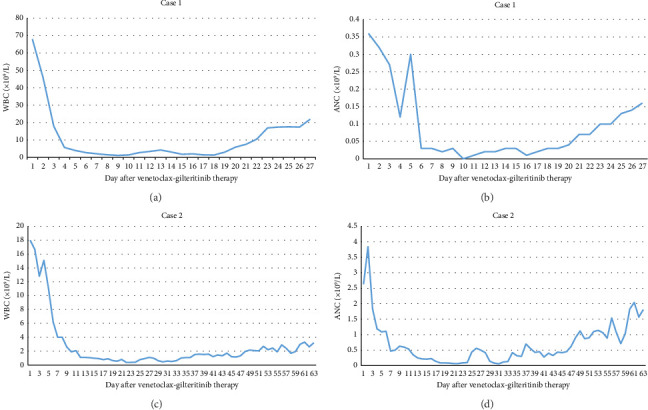
White blood cell (WBC) and absolute neutrophil count (ANC) numbers after gilteritinib–venetoclax treatment in Case 1 (a-b) and Case 2 (c-d).

**Figure 3:**
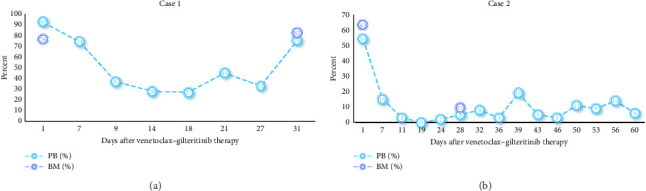
Proportion of blasts in BM and PB after gilteritinib–venetoclax treatment in Case 1 (a) and Case 2 (b). PB: peripheral blood; BM: bone marrow.

1. Case 1: After starting gilteritinib–venetoclax therapy, the patient experienced resolution of fatigue and nausea, indicating marked clinical improvement. Before initiation, hydroxyurea was given, reducing WBC counts from 137.78 × 10^9^/L to 67 × 10^9^/L. During the first 11 days of therapy, WBC counts decreased further to 1.13 × 10^9^/L but later increased to 20 × 10^9^/L (Figure 2). PB blasts fell from 92% to 27% within 18 days but rose to 79% by Day 28. Repeat BM evaluation on Day 28 showed no response, with blasts increasing from 76% to 82% (Figure 3), leading to treatment discontinuation.
2. Case 2: Following therapy, the patient reported relief of bone pain. PB blasts decreased from 54% to 0% within 24 days but later increased to 3%–19% during the second cycle (Figure 3). WBC counts declined from 18 × 10^9^/L to 0.39 × 10^9^/L in the first cycle and then rose to 3.2 × 10^9^/L in the second cycle. ANC improved from 0.07 × 10^9^/L to 2.04 × 10^9^/L during the second 28-day cycle (Figure 2). Hemoglobin and platelet counts showed no significant recovery. BM aspirates on Day 28 revealed a reduction in blasts from 63% to 9.5%, although CR was not achieved (Figure 3). Overall, blasts in BM and PB decreased by more than 50% compared with pretreatment levels.
3. Case 3: After starting therapy, nausea resolved and appetite improved. BM aspirates on Day 28 showed a decrease in blasts from 10% to 0%, with MRD negativity confirmed by flow cytometry. The patient achieved MLFS with persistent cytopenia.
4. Case 4: After one cycle, BM blasts decreased from 6% to 2%. The patient achieved MRD-positive CRi but declined further treatment due to financial constraints.

### 3.2. Survival

Case 1 received induction therapy with the HAA regimen. BM re-evaluation 21 days after completion showed 0% blasts, confirmed by flow cytometry, with persistent cytopenia, consistent with CRi. The patient died 24 days after completing one cycle of gilteritinib–venetoclax and the HAA regimen. Cases 2 and 3 died from severe infections related to prolonged neutropenic fever, bacteremia, and pneumonia after two cycles. Case 4 died 1 year after completing a single cycle.

## 4. Discussion

In this study, we evaluated the effectiveness of gilteritinib and venetoclax in four *FLT*3^WT^ MDS or AML patients with either relapsed disease (adaptive resistance) or refractory disease (primary resistance) after frontline treatment with hypomethylating agents or venetoclax-based combinations. Venetoclax has shown efficacy in AML and MDS when combined with hypomethylating agents or low-dose cytarabine [29]. However, resistance to current venetoclax-based regimens remains a major clinical challenge. Studies have reported that resistance to venetoclax–azacitidine therapy is associated with high *FLT3* signaling in *FLT*3^WT^ cells [30]. Thus, inhibition of *FLT3* signaling may provide therapeutic benefit for patients with either mutant or wild-type *FLT3* [31]. The mechanistic basis of activity also appears to involve targeting of AXL by gilteritinib, which does not depend on *FLT3*-*ITD*/*TKD* mutations or constitutive *FLT3* activation [25]. Another potential mechanism underlying clinical activity may involve induction of apoptosis, requiring both Bcl-2 inhibition by venetoclax and MCL-1 downregulation by gilteritinib [31]. Our findings suggest that gilteritinib combined with venetoclax may offer therapeutic benefit in R/R MDS/AML patients with *FLT*3^WT^, which represents a novel observation in this study.

In one case of R/R AML, gilteritinib combined with venetoclax produced a short-lived response, but subsequent treatment with the HAA (homoharringtonine, cytarabine, and aclarubicin) regimen achieved a marked therapeutic effect. Previous studies have shown that gilteritinib combined with homoharringtonine exerts a synergistic effect in *FLT3*-*ITD* AML cell lines [32]. Furthermore, gilteritinib combined with chemotherapy has been shown to reduce tumor volume more effectively than either agent alone in *FLT3*-*ITD* + AML cell lines and xenograft mouse models [33]. These findings suggest that combining gilteritinib–venetoclax with HAA chemotherapy may exert a synergistic effect in *FLT*3^WT^ R/R AML patients with in-frame bZIP-mutated *CEBPA*, further supporting the potential clinical efficacy of this therapeutic strategy.

It is reassuring that some efficacy of gilteritinib and venetoclax was also observed in an elderly patient with R/R *TP53*-mutated AML arising from preexisting MDS, who had complex karyotype alterations and a high *TP53* variant allele frequency, both associated with poor prognosis [34]. An in vitro study showed that gilteritinib was the most synergistic partner for venetoclax in the subgroup of patients with *TP53* mutation. This enhanced long-term efficacy may result from simultaneous targeting of BCL-2 by venetoclax and MCL-1 by gilteritinib, which increases early apoptotic responses in *TP53*-deficient cells [30]. Although co-occurring *PTPN11* mutations in this patient caused resistance to gilteritinib [35], gilteritinib–venetoclax therapy was more effective than other regimens, leading to a marked reduction of leukemic cells that lasted for 2 months. *TP53*-mutated AML is associated with poor response to hypomethylating agents and standard cytotoxic therapy [36–39]. The response to cytotoxic chemotherapy depends heavily on intact p53 function to induce apoptosis [40]; therefore, *TP53* mutations confer resistance to DNA-damaging agents [41]. The addition of venetoclax to standard regimens did not improve outcomes in *TP53*-mutated AML [42]. Although venetoclax-mediated apoptosis appears to be *TP53*-independent [43], combined p53 activation and BCL-2 inhibition act synergistically to induce lethality in leukemic cells [44]. Consistent with this, our *TP53*-mutated AML patient showed no response to low-intensity cytotoxic therapy with decitabine or venetoclax–azacitidine. A deeper understanding of the biology of *TP53* mutations and mechanisms of chemoresistance in AML, together with new strategies to improve survival, is urgently needed.

Our R/R MDS/AML patient with *ASXL1* mutation achieved MLFS with gilteritinib–venetoclax therapy but had no survival benefit. *ASXL*1^mut^ AML patients show higher CR/CRi rates after venetoclax–azacitidine induction, and this sensitivity has also been confirmed in vitro [45]. In R/R AML, *ASXL*1^mut^ patients also respond to venetoclax–azacitidine, but the MRD-positive rate and relapse rate remain high [42]. Furthermore, *ASXL*1^mut^ is not included in survival prediction in gene risk models after venetoclax–azacitidine treatment [46–48]. Based on the observed efficacy of gilteritinib and venetoclax in our R/R *ASXL*1^mut^ AML patient, the triple combination of gilteritinib, azacitidine, and venetoclax warrants further exploration for its potential to reduce relapse and prolong survival in *ASXL*1^mut^ AML.

Although gilteritinib–venetoclax therapy may not provide a cure for R/R AML/MDS patients, this pilot study suggests that it could improve general condition for palliative care and reduce tumor burden before allogeneic hematopoietic stem cell transplant (allo-HSCT). A limitation of this study is that qPCR or dPCR was not performed to assess the depth of molecular remission.

## Data Availability

All data used for this case report are contained within the article and its supporting files. These data are freely available for use without restriction.
